# A population-based study of the risk of osteoporosis and fracture with dutasteride and finasteride

**DOI:** 10.1186/s12891-018-2076-9

**Published:** 2018-05-22

**Authors:** Tony Antoniou, Erin M. Macdonald, Zhan Yao, Tara Gomes, Mina Tadrous, Joanne M.-W. Ho, Muhammad M. Mamdani, David N. Juurlink, for the Canadian Drug Safety and Effectiveness Research Network

**Affiliations:** 1 Department of Family and Community Medicine, St. Michael’s Hospital, 410 Sherbourne Street, 4th Floor, Toronto, ON M4X 1K2 Canada; 2 Li Ka Shing Knowledge Institute, St. Michael’s Hospital, 410 Sherbourne Street, 4th Floor, Toronto, ON M4X 1K2 Canada; 3 University of Toronto, 410 Sherbourne Street, 4th Floor, Toronto, ON M4X 1K2 Canada; 4 Institute for Clinical Evaluative Sciences, 410 Sherbourne Street, 4th Floor, Toronto, ON M4X 1K2 Canada; 5 Applied Health Research Centre, St. Michael’s Hospital, Toronto, ON Canada; 6 Department of Medicine, McMaster University, Hamilton, ON Canada; 7 Schlegel Research Institute for Aging, Waterloo, ON Canada; 8 King Saud University, Riyadh, Saudi Arabia; 9 Sunnybrook Research Institute, Toronto, ON Canada

**Keywords:** 5-alpha Reductase inhibitors/adverse effects, Osteoporosis/physiopathology, Dutasteride, Finasteride

## Abstract

**Background:**

Dutasteride is a potent inhibitor of 5-alpha reductase enzymes that reduces concentrations of dihydrotestosterone to a greater extent than finasteride. Whether this has adverse implications for bone health is unknown. We compared the risk of osteoporosis and fractures in older men treated with dutasteride or finasteride.

**Methods:**

We conducted a population-based retrospective cohort study with high-dimensional propensity score matching of Ontario men aged 66 years or older who started treatment with dutasteride or finasteride between January 1, 2006 and December 31, 2012. The primary outcome was a diagnosis of osteoporosis within 2 years of treatment initiation. A secondary outcome was osteoporotic or fragility fractures.

**Results:**

We studied 31,615 men treated with dutasteride and an equal number of men treated with finasteride. Dutasteride-treated patients had a lower incidence of osteoporosis than those receiving finasteride [2.2 versus 2.6 per 100 person years; hazard ratio (HR) 0.82; 95% confidence interval (CI) 0.72 to 0.93]. This effect was no longer statistically significant following adjustment for specialty of prescribing physician (HR 0.90; 95% CI 0.78 to 1.02)]. There was no differential risk of fractures with dutasteride (HR 1.04; 95% 0.86 to 1.25).

**Conclusions:**

Despite differential effects on 5-alpha reductase, dutasteride is not associated with an increased risk of osteoporosis or fractures in older men relative to finasteride. These findings suggest that dutasteride does not adversely affect bone health.

**Electronic supplementary material:**

The online version of this article (10.1186/s12891-018-2076-9) contains supplementary material, which is available to authorized users.

## Background

Osteoporosis is an under-appreciated cause of morbidity and mortality in men [1, 2]. Globally, one in three osteoporotic hip fractures occur in men, and a higher proportion of men than women die in the first year following a hip fracture, with mortality rates of 37.5 and 28.2%, respectively [3]. Evidence suggests that androgen deficiency contributes to bone loss and fracture risk in older men [4–7]. The observation that men with osteoporosis have lower dihydrotestosterone concentrations than men with normal bone mineral density suggests an important role of this androgen in bone homeostasis [8, 9]. Dihydrotestosterone is approximately ten times more potent than its precursor testosterone, and is the preferred ligand for androgen receptor transactivation [10]. Because 5-alpha reductases convert testosterone to dihydrotestosterone, inhibitors of these enzymes could conceivably predispose older men to osteoporosis and fractures [11].

Dutasteride and finasteride are 5-alpha reductase inhibitors that are equally effective treatments for benign prostatic hyperplasia [12]. Although considered clinically interchangeable, the two drugs differ in their spectrum of 5-alpha reductase inhibition. Finasteride is a selective inhibitor of the type 2 isoform of 5-alpha reductase, which is found predominantly in the prostate, while dutasteride additionally inhibits the more widespread type 1 isoform, which is the predominant 5-alpha reductase in osteoblasts [11–13]. Because dutasteride is both a more potent and less selective inhibitor of 5-alpha reductases, it reduces circulating serum dihydrotestosterone by 90 to 95%, compared with 60 to 70% for finasteride [11, 14, 15] In addition, dutasteride mediated-inhibition of 5-alpha reductase in osteoblasts could conceivably suppress local production of dihydrotestosterone [13]. These effects may have implications for bone health, particularly among older men, because bone loss accelerates rapidly after the age of 70 years [1]. However, whether dutasteride imparts a higher risk of osteoporosis and fractures in older men receiving 5-alpha reductase inhibitors is unknown.

Several studies have examined the effects of 5-alpha reductase inhibitors on bone mineral density. Although no significant changes in bone mineral density were observed in a one-year randomized trial of dutasteride and finasteride, the study was small and restricted to men aged 18 to 55 years [16]. Similar findings were observed in a small non-randomized study of men aged 60 to 78 years who were followed for up to two years after treatment initiation [17]. Observational studies have yielded inconsistent findings, ranging from no association between 5-alpha reductase inhibitors and bone disease to both a higher and lower risk imparted by these drugs [18–22]. Importantly, no observational study has specifically explored whether the available 5-alpha reductase inhibitors carry differential risks of osteoporosis or fracture in older men. This is important because benign prostatic hyperplasia is common in older men, 5-alpha reductase inhibitors are commonly prescribed for this indication, and male osteoporosis imparts a substantial burden on both health and society. We compared the risk of osteoporosis and fractures in older men commencing treatment with either dutasteride or finasteride. We hypothesized that, by virtue of more pronounced and widespread 5-alpha reductase inhibition, dutasteride might be associated with a heightened risk of these outcomes.

## Methods

### Study design

We conducted a population-based retrospective cohort study of Ontario men aged 66 years or older with no history of osteoporosis who commenced treatment with dutasteride or finasteride between January 1, 2006 and December 31, 2012. This study was approved by the Research Ethics Board of Sunnybrook Health Sciences Centre, Toronto, Canada.

### Data sources

We used the Ontario Drug Benefit (ODB) database to identify prescription drugs dispensed to Ontario residents aged 65 years or older, excluding the first year of eligibility for drug coverage (age 65) to avoid incomplete records. We obtained information regarding hospital admissions using the Canadian Institute for Health Information Discharge Abstract Database. We used the Canadian Institute for Health Information National Ambulatory Care Reporting System database to capture information regarding emergency department visits. We used the Ontario Health Insurance Plan database to obtain data regarding services provided by Ontario physicians and the Ontario Cancer Registry to exclude men with a history of prostate cancer. To identify patients with co-morbid illness, we used validated administrative registries to capture diagnoses of diabetes, hypertension, chronic obstructive pulmonary disease, asthma and congestive heart failure [23–27]. We determined physician speciality using the Institute for Clinical Evaluative Sciences Physician Database. Finally, we obtained basic demographic data from the Registered Persons Database. These databases, which are securely linked using unique, encoded identifiers and analyzed at the Institute for Clinical Evaluative Sciences (ICES, www.ices.on.ca). The use of these data sources and study methods are similar to those of our previously published studies exploring drug safety [28–30].

### Identification of cohort

We identified individuals prescribed dutasteride or finasteride using the ODB database, defining the index date as the date of first prescription for either drug. To restrict our analysis to new users of these drugs, we excluded individuals who received a prescription for either drug in the year before the index date. We deemed treatment continuous if a prescription was refilled within 1.5 times the days supplied by the preceding prescription. In both groups, we excluded individuals diagnosed with osteoporosis or a fragility fracture (see codes in Additional file 1 appendix) in the year preceding the index date, as well as those individuals who filled a prescription for oral bisphosphonates during this period. To avoid the potential confounding effects of prostate cancer and its treatments, we excluded men with a history of prostate cancer in the five years preceding the index date. We censored patients who discontinued treatment (defined by the date of the final prescription plus 1.5 times the prescription days’ supply), switched between study drugs, after two years of observation, at death, or at the end of follow-up (December 31, 2014), whichever occurred first.

To ensure the similarity of patients prescribed dutasteride and finasteride, we employed a matching algorithm using a high-dimensional propensity score approach to generate propensity scores for all patients in the cohort, as previously described [29, 30]. Variables forced into the propensity score included age, sex, Charlson comorbidity score, income quintile and long-term care status. We matched each patient treated with dutasteride to one patient treated with finasteride on propensity score (within 0.2 standard deviations), age at index date (within 2 years) and year of cohort entry.

### Outcomes

The primary outcome was a new diagnosis of osteoporosis, defined as any one of a physician visit, emergency department visit or hospital admission for osteoporosis (see supplemental appendix for International Classification of Diseases [ICD], Ninth and Tenth revision codes), or receipt of a prescription for an oral bisphosphonate. We considered only the first physician visit, hospital admission or emergency department visit for osteoporosis as a study outcome in patients who had multiple such encounters during the study period. In secondary analyses, we compared rates of osteoporotic or fragility fractures (Additional file 1 appendix for ICD-9 and ICD-10 codes). These codes have been previously validated for the diagnosis of osteoporosis in women, with accuracy measures exceeding 90% for discriminating osteoporosis from normal bone mineral density [31]. To test the specificity of our findings, we examined hospital visits for cataract surgery, since there is no plausible reason why the choice of dutasteride or finasteride would differentially influence this outcome.

### Statistical analysis

We calculated descriptive statistics for patients’ baseline demographic and clinical characteristics, and computed standardized differences to test for intergroup differences, with differences < 0.1 indicating good balance [32]. We conducted a matched Cox proportional hazards regression analysis for each outcome using finasteride as the reference group, given our prespecified hypothesis that dutasteride would be associated with an increased risk of osteoporosis and fractures. We adjusted all models for any baseline characteristics with a standardized difference exceeding 0.1 following propensity score matching. We did not conduct a sample size calculation; our study was population-based and we studied all eligible patients. All analyses were performed using SAS version 9.3 (SAS Institute, Cary, North Carolina).

## Results

During the six-year accrual period, we identified 31,615 individuals treated with dutasteride who were matched to an equal number of subjects treated with finasteride. Patients treated with dutasteride were followed for a median of 328 days (interquartile range 74 to 730 days), while those treated with finasteride were followed for a median of 313 days (interquartile range 64 to 730 days). Collectively, individuals in the cohort contributed a total of 65,804 person-years of follow-up. After propensity score matching, the two groups were highly similar in terms of demographics, medical illnesses and concomitant medications associated with osteoporosis and fractures (Table 1). However, patients differed with respect to the specialty of prescribing physician, with dutasteride-treated individuals more likely to have treatment prescribed by a urologist [16,862 (53.3%) versus 11,402 (36.1%)], whereas finasteride was more likely to be initiated by family physicians [13,553 (42.9%) versus 8924 (28.2%)] (Table 1).

**Table 1:** Baseline characteristics

Variable	Dutasteride users (*n* = 31,615)	Finasteride users (*n* = 31,615)	Standardized Difference
Age (median, IQR)	75 (71–81)	75 (71–81)	0.00
66–74	15,221 (48.1%)	15,150 (47.9%)	0.00
75–84	12,940 (40.9%)	13,031 (41.2%)	0.01
85+	3454 (10.9%)	3434 (10.9%)	0.00
Charlson Co-morbidity Index, No. (%) (2 years hospitalization data)			
No hospitalization	23,643 (74.8%)	23,547 (74.5%)	0.01
0	3365 (10.6%)	3241 (10.3%)	0.01
1	1740 (5.5%)	1785 (5.6%)	0.01
2 +	2867 (9.1%)	3042 (9.6%)	0.02
Number of prescription medications in previous year (median, IQR)	8 (4–11)	8 (4–11)	0.00
Residence in a long-term care facility, No. (%)	592 (1.9%)	724 (2.3%)	0.03
Medication use in previous 365 days, No. (%)			
Oral corticosteroid	1562 (4.9%)	1696 (5.4%)	0.02
Testosterone	357 (1.1%)	265 (0.8%)	0.03
Tenofovir	6 (0.0%)	14 (0.0%)	0.01
Thyroid hormone	2452 (7.8%)	2409 (7.6%)	0.01
Alpha-adrenergic blocker	5630 (17.8%)	6156 (19.5%)	0.04
Anticonvulsants	416 (1.3%)	477 (1.5%)	0.02
Tricyclic antidepressants	1167 (3.7%)	1213 (3.8%)	0.01
Selective serotonin reuptake inhibitors	2465 (7.8%)	2561 (8.1%)	0.01
Other antidepressants	13,798 (43.6%)	13,024 (41.2%)	0.05
Antipsychotics	859 (2.7%)	948 (3.0%)	0.02
Loop diuretics	3257 (10.3%)	3334 (10.5%)	0.01
Thiazide diuretics	5930 (18.8%)	5727 (18.1%)	0.02
Thiazolidinediones	628 (2.0%)	684 (2.2%)	0.01
Proton pump inhibitors	8439 (26.7%)	8386 (26.5%)	0.00
Benzodiazepines	5131 (16.2%)	4987 (15.8%)	0.01
Previous diagnoses, No. (%)			
Myocardial infarction	2325 (7.4%)	2440 (7.7%)	0.01
Diabetes	9531 (30.1%)	9647 (30.5%)	0.01
Asthma	3678 (11.6%)	3641 (11.5%)	0.00
Congestive heart failure	3675 (11.6%)	3855 (12.2%)	0.02
Chronic obstructive pulmonary disease	7717 (24.4%)	7601 (24.0%)	0.01
Dementia	3445 (10.9%)	3639 (11.5%)	0.02
Chronic kidney disease	1798 (5.7%)	1931 (6.1%)	0.02
Rheumatoid arthritis	94 (0.3%)	117 (0.4%)	0.01
Systemic lupus erythematosus	1888 (6.0%)	1954 (6.2%)	0.01
Medical conditions in previous 2 years, No. (%)			
Alcohol abuse	327 (1.0%)	384 (1.2%)	0.02
Stroke or transient ischemic attack	545 (1.7%)	583 (1.8%)	0.01
Prescribing physician, No. (%)			
Family physician	8924 (28.2%)	13,553 (42.9%)	0.31
Urologist	16,862 (53.3%)	11,402 (36.1%)	0.35
Other	1106 (3.5%)	1543 (4.9%)	0.07
Unknown	4723 (14.9%)	5117 (16.2%)	0.03
Specialist in preceding year, No. (%)			
Nephrologist	2249 (7.1%)	2484 (7.9%)	0.03
Endocrinologist	1973 (6.2%)	1615 (5.1%)	0.05
Geriatrician	1191 (3.8%)	1389 (4.4%)	0.03
Income Quintile, No. (%)			
1 (lowest)	5273 (16.7%)	5265 (16.7%)	0.00
2	6123 (19.4%)	6183 (19.6%)	0.00
3	6178 (19.5%)	6245 (19.8%)	0.01
4	6711 (21.2%)	6746 (21.3%)	0.00
5 (highest)	7217 (22.8%)	7062 (22.3%)	0.01
Missing	113 (0.4%)	114 (0.4%)	0.00

In the primary analysis, osteoporosis was diagnosed in 1569 individuals, with a lower rate observed among men prescribed dutasteride relative to finasteride [2.2 versus 2.6 per 100 person-years; hazard ratio (HR) 0.82, 95% confidence interval (CI) 0.72 to 0.93]. This difference was no longer statistically significant following adjustment for physician specialty (adjusted HR 0.90, 95% CI 0.78 to 1.02) (Table 2). In the secondary analysis, we found no difference in the risk of osteoporotic or fragility fracture between men prescribed dutasteride or finasteride (adjusted HR 1.04, 95% CI 0.86 to 1.25) (Table 2). As expected, we found no difference in cataract surgery between the two groups (HR 0.98; 95% CI 0.89 to 1.09).

**Table 2:** Risk of osteoporosis and fractures among patients treated with dutasteride or finasteride

	Number (%) of events in dutasteride treated individuals (n = 31,615)	Number (%) of events in finasteride treated individuals (n = 31,615)	Rate in dutasteride treated individuals (per 100 person years)	Rate in finasteride treated individuals (per 100 person years)	Unadjusted hazard ratio (95% confidence interval)^a^	Adjusted hazard ratio (95% confidence interval)^a^
Main outcomes						
Osteoporosis	729 (2.3%)	840 (2.7%)	2.2	2.6	0.82 (0.72 to 0.93)	0.90 (0.78 to 1.02)
Fracture	437 (1.4%)	463 (1.5%)	1.3	1.4	1.01 (0.85 to 1.21)	1.04 (0.86 to 1.25)
Tracer Outcome						
Cataract surgery	1487 (4.7%)	1551 (4.9%)	4.6	4.8	0.99 (0.90 to 1.08)	0.98 (0.89 to 1.09)

^a^Reference group is individuals treated with finasteride. Models adjusted for physician specialty

## Discussion

In this population-based study, we found no difference in the risk of fractures between older men treated with dutasteride or finasteride. Conversely, and in contrast to our study hypothesis, we found a lower incidence of osteoporosis in dutasteride-treated men compared with men treated with finasteride, but this was not statistically significant after adjustment for physician specialty. Our findings do not support a heightened risk of adverse bone outcomes among older men treated with dutasteride relative to finasteride, and that this drug can be used safely in this regard.

The finding that dutasteride did not increase the risk of adverse bone outcomes was unexpected in light of evidence that it inhibits dihydrotestosterone production to a greater extent than finasteride and that men with osteoporosis have lower levels of this androgen than men with normal bone mineral density [9, 15]. One possible explanation for this discordance is that dutasteride-mediated inhibition of osteoblast 5-alpha reductase activity is compensated for by higher levels of intracellular testosterone, which undergoes subsequent aromatization to estradiol [6, 13, 33]. This reasoning is supported by studies in men demonstrating better correlation between bone mineral density and serum estradiol rather than testosterone [33, 34]. Because it does not inhibit 5-alpha reductase in osteoblasts, this phenomenon would not be expected to occur with finasteride.

Another potential explanation for the lower crude rates of osteoporosis among dutasteride-treated men relates to the greater frequency with which this drug was prescribed by urologists relative to finasteride. Despite universal coverage of physician services, Ontario residents with high educational attainment have more contact with specialists and bypass primary care to reach specialists more often than those with lower education [35]. Extending these findings to our study, it is possible that the lower incidence of osteoporosis among dutasteride recipients reflects in part greater knowledge about osteoporosis prevention and a healthy user effect, an assertion supported by the loss of a statistically significant association between dutasteride and osteoporosis after adjustment for physician specialty. Detection bias may also account for this finding because osteoporosis screening is more likely to be undertaken by family physicians than urologists, with the former ordering approximately 80% of bone mineral density tests in Ontario [36]. Importantly, however, rates of osteoporosis screening among men at high risk for the disease consistently lag far below those of women. In Ontario, age-standardized rates of bone mineral density testing within 6 months of a fracture were 8 per 100 men, compared with 16.1 per 100 women, in 2009/2010 [37]. Similarly, only 11% of Ontario men between the ages of 68 to 70 who had never previously undergone bone mineral testing received such a test in 2009/2010, compared with over 40% of Ontario women in this same high-risk group [37]. These findings are supported by those of a Canadian cohort study in which 90% of men with fragility fractures remained undiagnosed and untreated for osteoporosis despite participation in a five-year study with the potential to raise awareness of the disease among participants and their physicians [38]. Interventions to promote osteoporosis diagnosis and treatment among men are warranted.

Our study builds on previous observational studies examining the association between adverse bone outcomes and 5-alpha reductase inhibitors in several ways. First, ours is the first study to specifically compare the risk of osteoporosis and fractures between dutasteride and finasteride. Previous studies have risk estimates for 5-alpha reductase inhibitor exposure relative to a control group of untreated men [18–22]. Second, we generated additional data regarding the risk of osteoporosis in men treated with 5-alpha reductase inhibitors, an outcome examined in only one case control study with 47 osteoporosis patients exposed to one of these drugs [21]. Finally, we were able to demonstrate the important confounding role of physician specialty in the association between adverse bone outcomes and 5-alpha reductase inhibitors. Specifically, the finding that adjustment for physician specialty reversed the association between dutasteride and osteoporosis suggests that future studies of these drugs consider accounting for health service delivery and other indicators of a potential healthy user effect.

Our study has some limitations. We used administrative data rather than measurement of bone mineral density to identify patients with osteoporosis, and codes used for ascertaining osteoporosis have not been validated among older men. We had no access to relevant clinical variables such as smoking history, alcohol use, body mass index and use of over the counter calcium and vitamin D supplementation. However, these limitations apply equally to both groups of patients. We followed patients for two years from the time of drug initiation; it is possible that additional outcomes may have been observed with longer follow-up. We selected a two-year follow-up because of the advanced age of our cohort, reasoning that prostate cancer and frailty could emerge as important confounders with a longer follow-up period. In addition, we had no information regarding medication adherence. Finally, because we conducted our analyses in men aged 66 years and over with no prior history of osteoporosis or fractures, our findings may not be applicable to younger patients and those with pre-existing bone disease.

## Conclusions

We found no difference in the incidence of a new diagnosis of osteoporosis among older men treated with either dutasteride or finasteride. Similarly, we found no differential risk of fractures with dutasteride. These findings provide a measure of reassurance that widespread inhibition of 5-alpha-reductase does not negatively affect bone health in older men, and that dutasteride can be safely used in this regard.

## Additional file

Additional files 1: Online Appendix ICD-9 and ICD-10 Codes For Outcome Ascertainment. Administrative codes for osteoporosis and fractures. (DOCX 13 kb)

## Abbreviations

CI: Confidence interval
HR: Hazard ratio
ICD: International Classification of Diseases
ICES: Institute for Clinical Evaluative Sciences
ODB: Ontario Drug Benefit
