# Supplementary material for: A population-based study of the risk of osteoporosis and fracture with dutasteride and finasteride
Source: BMC Musculoskelet Disord. 2018 May 22;19:160. doi: 10.1186/s12891-018-2076-9 (PMC5964967; doi:10.1186/s12891-018-2076-9)
Supplement: Supplementary file 1 — Online Appendix ICD-9 and ICD-10 Codes For Outcome Ascertainment. Administrative codes for osteoporosis and fractures. (DOCX 13 kb) [file 12891_2018_2076_MOESM1_ESM.docx]

Online Appendix: ICD-9 and ICD-10 Codes For Outcome Ascertainment

| Outcome | ICD-9 | ICD-10 |
| --- | --- | --- |
| Osteoporosis | 733 | M81 |
| Fractures | 805, 812, 813, 814, 820, 821 | M80 |
